# Intracranial recordings in humans reveal specific hippocampal spectral and dorsal vs. ventral connectivity signatures during visual, attention and memory tasks

**DOI:** 10.1038/s41598-022-07225-0

**Published:** 2022-03-03

**Authors:** João Castelhano, Isabel Duarte, Inês Bernardino, Federica Pelle, Stefano Francione, Francisco Sales, Miguel Castelo-Branco

**Affiliations:** 1 ICNAS, University of Coimbra, Polo 3, Azinhaga de Santa Comba, Celas, 3000-548 Coimbra, Portugal; 2 CIBIT, Faculty of Medicine, University of Coimbra, Coimbra, Portugal; 3 Claudio Munari Epilepsy Surgery Center, Niguarda Hospital, Milan, Italy; 4 Epilepsy Unit, CHUC, Coimbra, Portugal

**Keywords:** Neural circuits, Cognitive neuroscience

## Abstract

Invasive brain recordings using many electrodes across a wide range of tasks provide a unique opportunity to study the role of oscillatory patterning and functional connectivity. We used large-scale recordings (stereo EEG) within and beyond the human hippocampus to investigate the role of distinct frequency oscillations during real-time execution of visual, attention and memory tasks in eight epileptic patients. We found that activity patterns in the hippocampus showed task and frequency dependent properties. Importantly, we found distinct connectivity signatures, in particular concerning parietal-hippocampal connectivity, thus revealing large scale synchronization of networks involved in memory tasks. Comparing the power per frequency band, across tasks and hippocampal regions (anterior/posterior) we confirmed a main effect of frequency band (p = 0.002). Gamma band activity was higher for visuo-spatial memory tasks in the anterior hippocampus. Further, we found that alpha and beta band activity in posterior hippocampus had larger modulation for high memory load visual tasks (p = 0.004). Three functional connectivity task related networks were identified: (dorsal) parietal-hippocampus (visual attention and memory), ventral stream- hippocampus and hippocampal-frontal connections (mainly tasks involving face recognition or object based search). These findings support the critical role of oscillatory patterning in the hippocampus during visual and memory tasks and suggests the presence of task related spectral and functional connectivity signatures. These results show that the use of large scale human intracranial recordings can validate the role of oscillatory and functional connectivity patterns across a broad range of cognitive domains.

## Introduction

It is well-known that the hippocampal formation plays a critical role in a wide set of cognitive tasks, particularly in the memory encoding stage of verbal and visuospatial information^1,2^. Functional specialization of distinct subregions within hippocampus and the role of distinct oscillatory patterns and synchrony remain unclear but the separation of functions of anterior and posterior regions of the hippocampus is well-recognized^3–6^. The posterior hippocampus has been shown to be involved in spatial memory and navigation and responses in this region index stimulus familiarity^7^. Such functions seem to be supported by strong connectivity with the thalamus, parietal, posterior cingulate and occipito/temporal cortices^8^. The anterior hippocampus mediates more complex memory functions and novelty processing^7,9,10^. It sends and receives input from areas such as the dorsal and medial prefrontal cortices^7,8,11^. Although many studies have addressed the functions of hippocampus and its sub regions using fMRI (e.g.^12^) they lack temporal resolution to critically test the role of functional connectivity at the millisecond time scale. The advantage to investigate the functional role of high and low frequency activity patterns using invasive recordings is well recognized (e.g.^13–18^) although previous studies focused on very specific and narrowly defined tasks. The investigation of cognitive performance using a broad set of tasks at time scales relevant to behavior may shed light on the functional role of hippocampal activity in particular if one addresses functional connectivity and not just patterning across distinct frequency bands. Invasive large scale recording approaches, focusing on the functional connectivity of the hippocampus, across a broad range of tasks, might constitute a relevant approach to disentangle the functional role of task related temporal activity patterns. However, there is no direct electrophysiological evidence showing task related hippocampal oscillatory activity and large scale functional connectivity in humans performing cognitive evaluation tasks in real clinical settings.

We addressed this question by the analysis of task related oscillatory and synchronization patterns recorded directly from the hippocampus and a large set of brain regions of epileptic patients with medication resistant epilepsy in whom stereotactic electrodes had been implanted in the hippocampus for pre-surgical evaluation. Patients submitted to stereo-electroencephalography (sEEG) have a 3D array of electrodes implanted in different areas of their brain for localization of seizure foci^19–21^. Due to their higher spatial resolution, sensitivity and signal-to-noise ratio these data provide a unique opportunity to study the dynamics of brain oscillations^22,23^. Previous reports found a crucial role of hippocampal slow EEG frequencies during sleep in the memory consolidation processes^24^. In the same line, our previous work and others demonstrated a link between theta oscillations and self-motion information and spatial-memory^25,26^ and an association between high-frequency patterning and recognition memory^17,23^. In spite of the identified links between activity across different frequency bands and distinct brain functions^27,28^, little is known in relation to their relevance to network organization, as defined by functional connectivity approaches.

Here, we aimed at investigating from the electrophysiological point of view the human hippocampus and a large set of brain regions oscillatory responses to cognitive evaluation tasks. We aim to identify task relevant spectral and synchrony signatures within the hippocampus. To this end we take advantage of concomitant neuropsychological assessment. We hypothesized that distinct cognitive tasks (with different cognitive and memory load) should be distinctly related to the power and synchrony of the various frequency bands (alpha, beta, low and high gamma) in the hippocampus and the regions connected to this structure^29^.

In this study, we show that alpha and beta bands activity in posterior hippocampus are modulated by the cognitive load of the task (lower power for simpler visuoconstructive tasks e.g. copy Rey figure). Moreover, gamma oscillations are differentially modulated by tasks type in the anterior hippocampus. We identified distinct task related connectivity patterns, in particular concerning parietal-hippocampal connectivity. Overall, our results provide evidence for the critical role of task related spectral signatures and connectivity patterns stemming from the hippocampus.

## Results

### Experiment design and electrode localization

**Figure 1:**
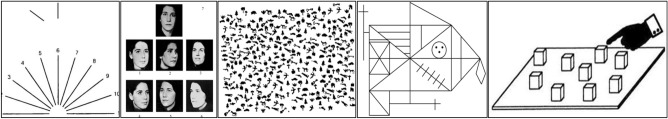
Example of cognitive tasks. Patients performed a full set of tasks, including visuo-spatial memory and visual tasks. From left to right: Benton judgment of line orientation; Benton face recognition; visual selective attention—Bells test; Rey complex figure, (copy/encoding and recall); Corsi block-tapping test, a visual spatial memory test and long term memory Corsi Supraspan). Please note that these faces are from the publicly available Benton facial recognition test (https://pubmed.ncbi.nlm.nih.gov/29549569/).

We performed invasive recordings from eight human patients with intracranial depth electrodes. We examined neuronal oscillatory activity and synchrony while patients were performing classical cognitive tasks often used in neuropsychological assessment encompassing visuo-spatial memory, visual perception and search (Fig. 1, for details see “Methods”).

Tables 1 and 2 summarize the behavioral outcome of each task, contacts location and further demographic and clinical information of each patient.

**Table 1:** Demographic and neuropsychological characterization.

	Subj 1	Subj 2	Subj 3	Subj4	Subj 5	Subj 6	Subj 7	Subj 8	
Chronological age	51	39	23	30	34	26	45	35	
Education level (years)	8	17	12		18	13	13	9	
Gender	F	M	M	M	F	F	M	M	
**Reference value**									
JLO	24	28	25	24	25	27	24	17	Cut-off: 19
Benton face recogniton	43	45	49	50	50	52	43	39	M: 41–54
RCF—copy Rey	11.5	32	36	33	34	29	28	26	M:32, SD:1.8
RCF—recall Rey	14.5	25	18	24.5	22	24	12.5	15	M:22.9, SD:2
Bell’s test—selective attention	33	34	35	35	35	32	28		M: 35
Corsi—visuospatial memory	2	4	2	4	4	3	3	2	Standardized scores
Corsi Supraspan—long term mem	0	4	3–4		3	4	0		Standardized scores

Raw scores as well as the reference values are presented for the neuropsychological tests except for the Corsi test in which the standardized scores are shown (0 = severe impairment; 1 = inferior to the mean; 2–3 = mean values; 4 = above percentile 50).

*Subj* subject, *JLO* judgment of line orientation, *RCF* Rey complex figure, M mean, *SD* standard deviation.

**Table 2:** Patients clinical characterization and acquisition details.

	Subj 1	Subj 2	Subj 3	Subj4	Subj 5	Subj 6	Subj 7	Subj 8
Age of seizure onset	22	12	8	24	11	18	22	14
sEEG date	2013	2013	2012	2015	2013	2015	2015	2015
Electrodes related to seizure	Right centro-temporal	Left occipito-temporal	Right fronto-temporal	Temporal neocortical	Left temporal and centro-temporal	Right temporo-parietal	Right occipito-temporal	Right fronto-polar
Hemisphere	Right	Left	Right	Right	Left	Right	Right	Right
Locs hip ant	B2 B3 B4 B5	B2 B3 B4 B5 B6		B2 B3 B4 B5	B2 B3 B4 B5 B6	B1 B2 B3 B4	B2 B3 B4 B5 B6	52–56
Locs hip post	C3 C4 C5	C3 C4 C5 C6	C2 C3 C4 C5	C3 C4 C5	C2 C3 C4 C5	C3 C4 C5	C4 C5 C6	

	JLO	BentonFace recog	Recall Rey	LongTerm mem	Visuospatialmem	Sel.Attention	Copy Rey	
Accepted trials Avg. (SEM)	112 (22)	99 (37)	48 (7)	116 (34)	71 (10)	52 (22)	68 (20)	
Rejected trials Avg	37	60	11	55	17	37	33	

Rejected trials represent the trials discarded to epileptic artefact rejection.

*Subj* subject, *Avg* average, *SEM* standard error of the mean.

We recorded these intracranial data while patients were task engaged (example video available as supplementary material 1, (patient wearing a cap and positioned to prevent identification). This real-time scenario in long recording periods provides a unique opportunity to evaluate the brain in a naturalistic manner, unlike other less ecological and more rigid experimental approaches^26^.

**Figure 2:**
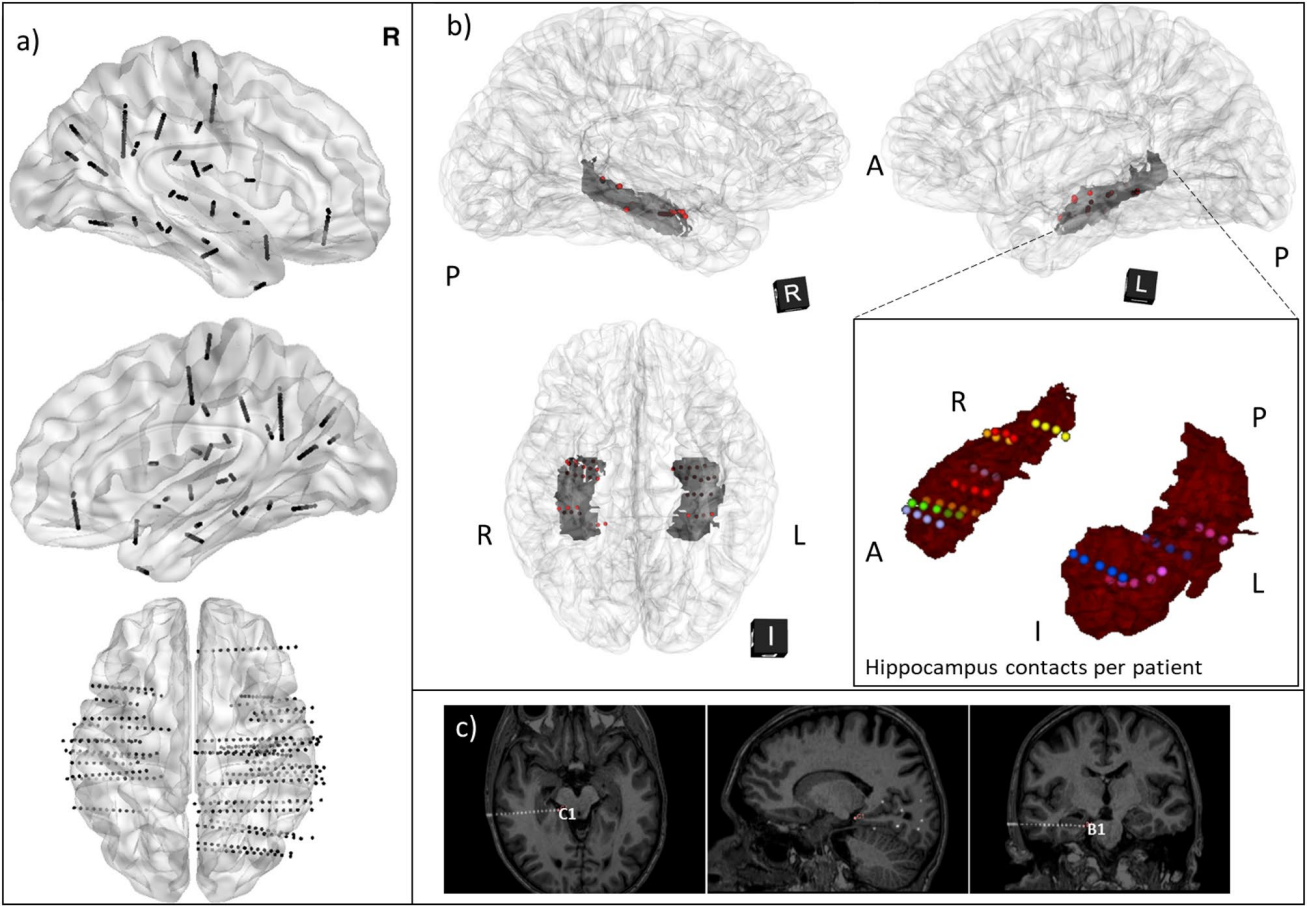
Localization of the implanted electrodes and contacts within hippocampus. **(a)** Example of all the contacts for four patients (to prevent cluttering we did not plot all subjects’ positions). All locations were co-registered on a brain in Talairach space. **(b)** 3D reconstruction of the contacts position in the standard brain. Contacts within the hippocampus of each individual participant were transformed to the standard brain and are represented as red spheres and with different colors per patient in the hippocampus volume reconstruction. Dark grey represents the volumetric reconstruction of the hippocampus. **(c)** Example of the locations of two contacts (B1 and C1) used during our data analysis. In **(c)**, contacts are represented in their native image space. See the supplemental material 2 for a video of those hippocampus locations.

Depth electrode localization was determined based on the co-registered pre and post-implantation imaging procedure (CT and MRI). Localization of each electrode and contacts was visually confirmed by an experienced neurosurgeon (S.F.). In all subjects, there were at least four contacts located in the hippocampus. See Fig. 2 for a representation of all implanted electrodes and a three dimensional reconstruction showing all contacts within the hippocampus.

### Time-activity patterns in hippocampus for distinct cognitive tasks

**Figure 3:**
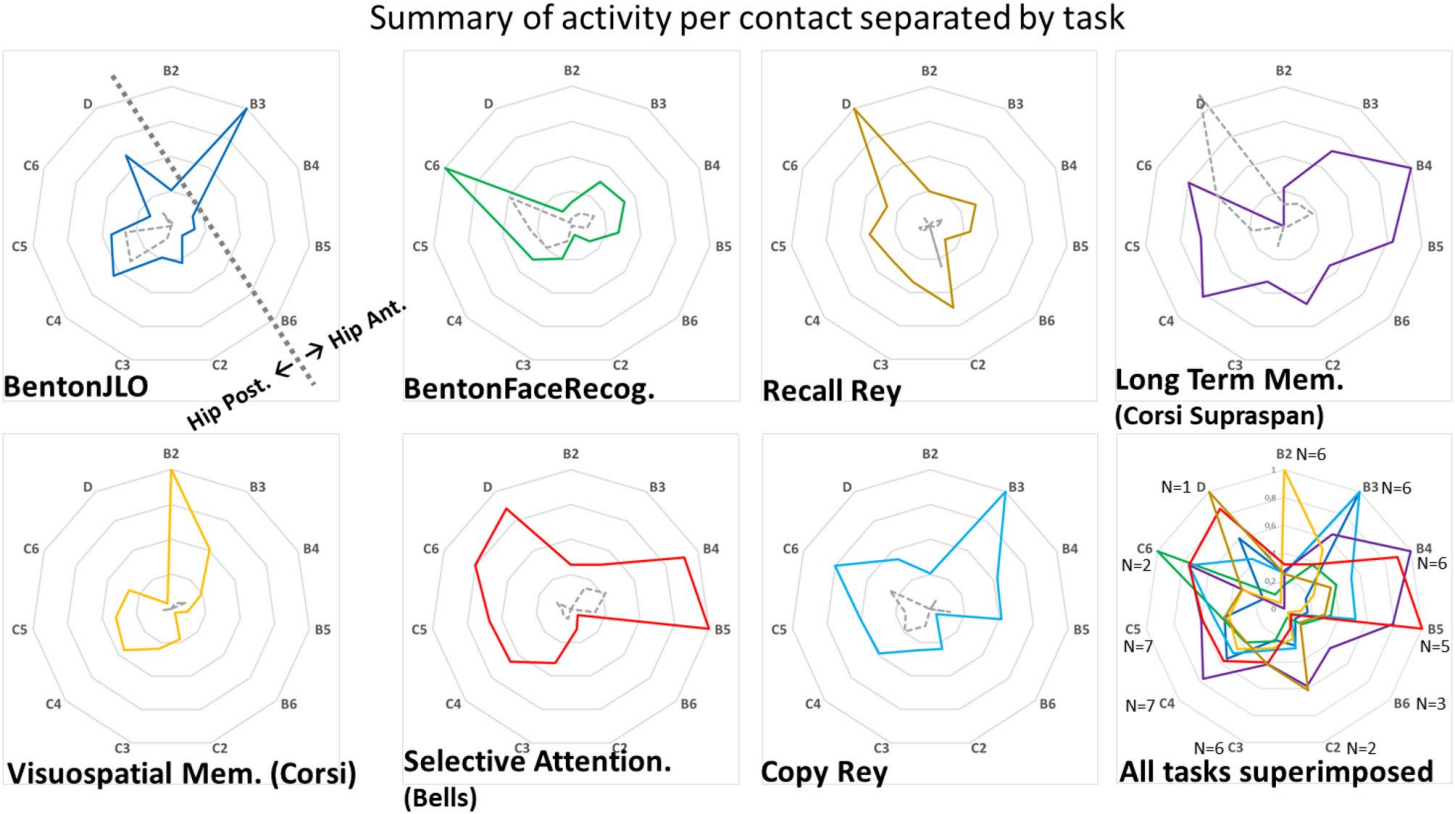
Time domain activity variation per task within the human hippocampus (anterior and posterior partitions) reveals distinct patterns of specificity across tasks. Time activity magnitude peaks per hippocampus individual contact and task condition are represented. Time-activity peaks for the different tasks were defined as the max magnitude 2SD above mean baseline magnitude. We then count the number of peaks over threshold per task and statistical analysis revealed significant task activation above the baseline (dotted grey line in the plots). Plots represent normalized data and each task maximum magnitude (scale is 0:0.25:1 for the individual plots and 0:0.2:1 for the superimposed plot). The last panel represent all tasks overlaid for sake of visual comparison and report the number of subjects (N) contributing to each contact results. Note that distinct contacts within hippocampus have high preference for different tasks.

To assess the prediction that distinct tasks have different spectral signatures within the hippocampus, we analyzed data containing different types of task (visuo-spatial memory, and visual attention). After baseline correction for the time window preceding each task, we identified the peaks defined as the max magnitude 2 Standard Deviation above mean baseline magnitude, to derive a count measure of the number of task related peaks that passed the Z score threshold. We found task related patterns of activation for the distinct individual contacts. For sake of simplicity, these results are summarized in Fig. 3 by showing the activity per condition and contact in radial plots. We expected to find an effect of memory load as well as task dependent effects in the anterior and posterior hippocampus. Differences between Anterior Hippocampus and Posterior Hippocampus were indeed found (e.g. recall of the Rey figure specifically recruited the posterior hippocampus unlike the simple copy of the same figure). Significance of neuronal response patterns (number of peaks above threshold) suggests the involvement of the hippocampus in the following tasks: (Wilcoxon non-parametric Z scores and p values for Task activation): Benton JLO, Z = 3.99 p = 0.000067; Benton Face Z = 5.29 p = 1.2033E−7; Copy Rey Z = 3.83 p = 0.00013; Long Term Memory (Corsi Supraspan) Z = 4.358 p = 0.000005; Visuo Spatial Memory (Corsi) Z = 5.04 p = 4.6049E−7; Visual Selective Attention (search) Z = 5.34 p = 9.4624E−8; Recall Rey Z = 5.82 p = 5.7382E−9.

### Intracranial recordings reveal task dependent oscillatory activity within human hippocampus

**Figure 4:**
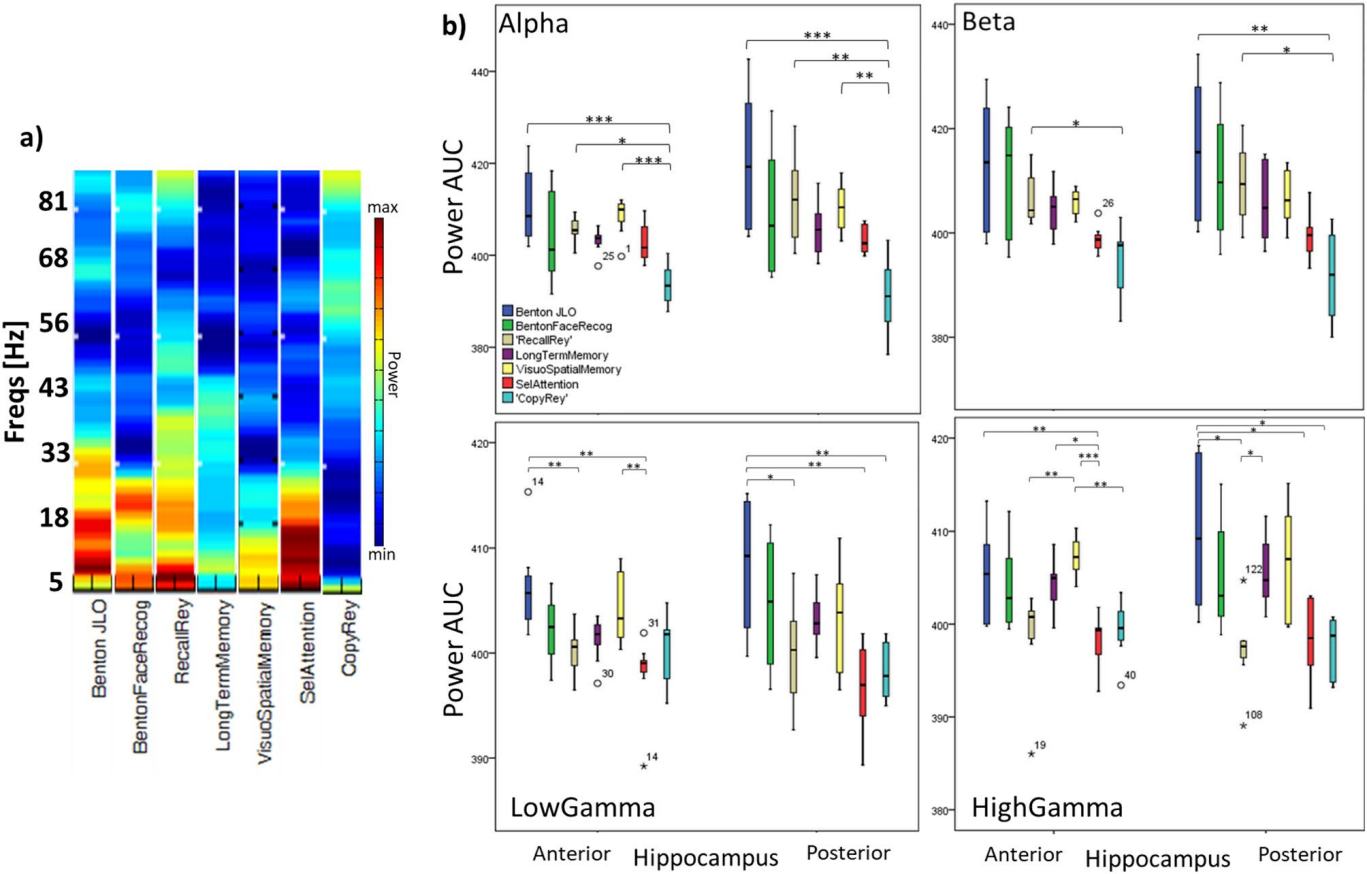
Trial-by-trial average power patterns at different frequencies and task conditions. The trim-mean power at different frequency bins was computed across whole task duration. We computed the results separately for each contact and averaged them per hippocampal region. (**a)** The power per task is shown. **(b)** Boxplots represent the area under the curve for the alpha, beta, low gamma and high gamma frequency bands. Plots show the data per task separated by the anterior and posterior regions of the hippocampus. Note the task dependent power values and the downward trend as simple visual demands dominate (AUC—area-under-the-curve). Significant differences are marked with * as follows: *** p < 0.000, **p < 0.007, *p < 0.04.

Neuronal networks typically exhibit activity in distinct oscillatory bands with distinct locations and putative roles in the brain^30^. We thus explored neuronal activity patterns in the time–frequency domain^31–34^. We found different activity modulation patterns as a function of task as summarized in Fig. 4. We investigated patterns of change across four main bands (alpha, beta, low and high gamma). Statistical analysis (Friedman test) comparing the power per frequency band, tasks and hippocampal regions (anterior/posterior) confirmed a main effect of frequency band (posterior Hippocampus: p = 0.002 for Rey recall and visual selective attention tasks; Anterior Hippocampus: p = 0.044 for Benton Face Recognition, p = 0.005 for Copy Rey, p = 0.032 for visuospatial memory (Corsi), p = 0.000 for visual selective attention and recall Rey tasks).

Further analysis confirmed that hippocampal regions activated in a task and frequency dependent manner. In the lower frequency bands, we found mainly differences between tasks involving higher memory and/or executive load and tasks requiring simple visual analysis (Copy Rey and Simple visual search) (Fig. 4). Accordingly, we found a pattern of larger activation in alpha and beta bands in posterior hippocampus for tasks with larger cognitive load as compared to tasks with simpler visual demands (lower power for gradually simpler visual tasks; Spearman correlation rho = −0.248 p = 0.004). For example, visually guided copy of the Rey figure task yielded lower alpha and beta power when compared to Recall (F = 3.31 p < 0.019), Benton JLO (F = 4.55 p = 0.007) and visuospatial memory (Corsi) (F = 4.65 p = 0.007) tasks.

Power at gamma frequencies was higher for demanding visuo-spatial memory and Judgement of line orientation (JLO) tasks, in particular in the anterior hippocampus (p = 0.007). High gamma activity at posterior Hippocampus was mainly related to JLO tasks. Our results are consistent with the notion that neural oscillatory activity at different frequency bands changes in a task and hippocampal region dependent manner.

### Functional connectivity stemming from the hippocampus is task dependent

**Figure 5:**
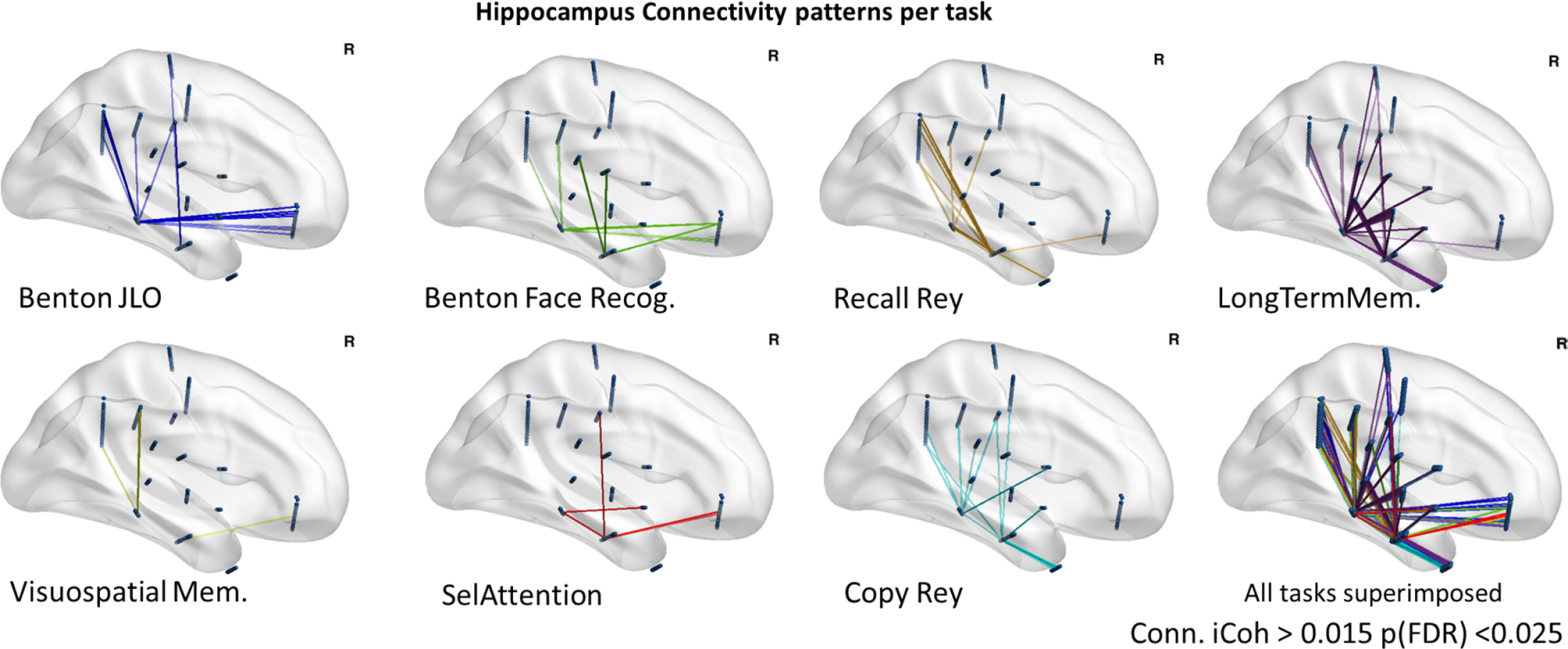
Graphical representations of contact pairs showing significant connectivity. Data plotted are an example from one subject (Subj3) for all the different tasks, iCoh (p(FDR) < 0.025); each map represent the links between hippocampal regions and other brain regions during the correspondent task. Note the task dependent connectivity patterns, in particular the occipital and parietal-hippocampal connectivity, generalizable for 6 patients. For the sake of simplicity, connectivity map plots are separated by task and overlaid in the last panel. See more individual plots in supplementary material 3.

To understand the functional connectivity associated with these memory related tasks, we computed the data imaginary coherence (iCoh; a measure of synchrony representing connectivity between brain regions). We found task dependent patterns of connectivity between the hippocampus and different brain regions. Accordingly, iCoh measures revealed significant differences in sEEG functional connectivity networks between conditions (p(FDR) < 0.025). Most relevant results are illustrated in Fig. 5 for a representative subject, and highlight neural synchrony patterns that match predicted functional relationships across regions. We identified functional connectivity networks involving parietal regions for the tasks requiring visual processing tasks (this effect could be detected in 6 patients with corresponding electrode locations). The hippocampal connectivity with ventro-temporal areas is particularly increased for tasks requiring complex visual form processing, in particular during the Benton Face task as expected (p(FDR) < 0.025). Furthermore, the anterior hippocampus show higher connectivity to pre-frontal and central areas while in the other hand the posterior hippocampus shows larger connectivity with parietal and temporal regions, in a task dependent manner.

## Discussion

We demonstrate here that high-resolution recordings directly from human hippocampus^15,35^ can be used to map task related activity, and in particular task related oscillatory patterns evoked by visual tasks with or without memory load. To our knowledge, this is the first study including sEEG analysis of oscillatory activity and of functional connectivity with wide spatial coverage of patients performing distinct cognitive tasks for long periods. By analyzing electrical activity in the time and frequency domains^2,18^, we found task dependent frequency and synchrony patterns that shed a new light on hippocampus function and connectivity^3^. We found that although the observed patterns are consistent with evidence suggesting that memory traces are organized in a topographical manner^3,7^ but also shows that even tasks without memory constraints activate the hippocampus.

This work is quite distinct from previous studies that have used short-lived visual stimuli and task designs^21,36,37^. We have used an approach that takes advantage of the invasive large scale recordings while patients perform distinct visual and/or memory related tasks for long periods of time. We were able to visualize patterns of activation and functional connectivity of the human hippocampus during real-life recordings and this provides a new framework to understand the role of the hippocampus in cognition^11,26^.

We demonstrated that at any given location in the hippocampus there is specific preference for task type. During cognitive tasks, brain shows changes in power in specific frequency ranges^2^. We found e.g. power at gamma frequencies was higher for tasks with high memory and/or attentional load, in particular in the anterior hippocampus. Simpler tasks such as copy Rey Figure showed the lowest modulation profile in the hippocampus. The posterior hippocampus showed a significant largest modulation as a function of visual task complexity, in particular in the alpha and beta ranges. Beta oscillations in the anterior hippocampus were less task discriminative than gamma oscillations. Our approach using state of the art methods proved it is possible to analyze comprehensively neural activity evoked by long lasting cognitive tasks^2,21,37^ realized in standard clinical settings.

Importantly, we were able to investigate the functional connectivity of the networks involved in those tasks and investigate their task dependence, and highlight relevant processing streams, such as parietal-hippocampal connectivity in tasks requiring high visual attentional and/or memory load.

In general, we found that the anterior hippocampus shows higher connectivity to frontal and central areas while in the other hand the posterior hippocampus is connected within a network comprising parietal and to some extent temporal regions, in a task dependent manner. These results are consistent with known posterior and anterior hippocampus anatomical connectivity^7,8,11^. In spite of the limitations implied by the clinical settings the large amount of subject specific contact locations, renders this a quite important dataset (with more than 1200 locations recorded in the brain) that leverages to the millisecond time scale information from brain connectome projects. Our results provide a framework for future studies of the interplay of task related oscillatory modulations in the hippocampus role and functional connectivity^11,15^.

This work basic identified three functional sets of task related connections: (dorsal) parietal-hippocampus, ventral stream- hippocampus and hippocampal frontal connections providing a functional connectivity perspective at the millisecond time scale. The translation the approaches used here to understand brain reorganization in the clinical setting^22^ remains an open possibility and the present work is a promising step to the study of brain connectivity during real-life tasks.

## Methods

### Subjects and neuropsychological tasks

Patients with medication-resistant epilepsy had been submitted to stereo-electroencephalography (sEEG) with a 3D array of electrodes implanted in different areas of their brain for localization of seizure foci for possible surgical resection^19–21^. Electrodes were implanted for diagnostic purposes using a computerized tomography-based stereotactic insertion technique^20,38^ and the Hospital Niguarda Milano team collected data. The institutional review boards of the Hospital Niguarda Milano (Comitato Etico Milano Area 3) approved the research protocol. Research was performed in accordance with all relevant guidelines/regulations, in accordance with the Declaration of Helsinki. An informed consent was obtained from the subjects and all the data were acquired and analyzed following the European data protection rules.

Eight patients (age: 33.5 ± 10.9 years; 3 Female) had implanted depth electrodes with contacts reaching the hippocampus region. The placement of the electrodes was strictly determined by the clinical criteria and patient needs. Each patient underwent then a comprehensive neuropsychological evaluation by an experienced psychologist and simultaneous intracranial data were recorded while they performed a set of cognitive tasks (Fig. 1). We recorded the invasive data continuously and synchronized with video recording of the assessment sessions. The neuropsychological evaluation included: Benton Judgment of line orientation (matching a pair of angled lines with a set of lines representing different angles—visuospatial perception) (JLO:^39^); Benton face recognition (matching a target face with a set of faces—visual perception and recognition) (BFRT;^40^); the Bells test (cancellation task—selective attention)^41^; Rey Complex Figure (copy and recall tasks—visuoconstructive and visual memory, respectively) (RCF;^42^); Corsi Block-tapping test^43^ (tapping a sequence of blocks in the same or in the inverse order as the examiner showed—visuospatial working memory and supraspan—Long term memory). Video recordings were used to define the beginning and end of each task. An experienced psychologist and a biomedical engineer performed that job independently. We only included for the analysis of each task the patients that were able to perform the task within the normal scoring range (see Table 1 for details on behavioural outcomes).

### Depth electrode localization

Electrodes were localized in each patient using co-registered pre-implantation and post-implantation structural T1-weighted MRI scans and computed tomography scans^20,44,45^. For each participant, the post- and pre-implantation scans were co-registered using a nine-parameter rigid body transformation (Slicer 4.3.1 software with default parameters). A standard hippocampus mask was then used for help determining exact electrode locations. This allowed for visualization and accurate identification of electrode/contact locations in each participant’s space. An experienced neurosurgeon (author S.F.) then selected and labeled the hippocampus contacts of each participant in a semi-automated manner, guided by the anatomical atlas of the hippocampus. Table 2 summarizes the sEEG acquisition and clinical characterization of each participant.

### Data collection and pre-processing

Intracranial EEG data were acquired using a Nihon Kohden EEG2100 system, acquiring 192 channels at 1000 Hz sampling frequency per subject. Patients are chronically recorded (with overall more than 1200 locations recorded in the brain) and the administration of the cognitive battery described above is performed during 1 or 2 session of 1 to 2 h each, depending on the patient's characteristics. All the data were analysed in MATLAB combined with open source toolboxes (EEGLAB^46^; HERMES^47^; BrainNet Viewer^48^). Neuronal recordings were band-pass filtered from 0.1 to 350 Hz using a zero phase delay finite impulse response (FIR) filter with Hamming window for subsequent analyses.

### Time–frequency analyses of recordings from the hippocampus

We divided the data into blocks containing different tasks (Rey Figure: copy and recall; Benton tasks: JLO and Face Recognition; Corsi: visuospatial and long term memory; and Bells test: visual selective attention). We hypothesized that during cognitive tasks electrical activity in the hippocampus shows changes in power and synchrony in specific frequency ranges. We then performed Time–frequency analysis between 5 and 80 Hz as described elsewhere^28,49^ in 4 s epochs. We defined the baseline time-window as the blocks between tasks (minimum 2 min). There were no seizures recorded in any of the epochs, all epochs were visually inspected for artifacts (e.g., epileptiform spikes) and any signal blocks with artifacts were removed from analysis (Table 2 summarizes the number of trials used/rejected per condition). To extract separate frequency components and examine neuronal responses, induced oscillations were detected by frequency band of interest (Theta 4–8 Hz, Alpha 8–12 Hz, Beta 15–25 Hz, low-Gamma 30–45 Hz, high gamma 55–80 Hz) in the hippocampal contacts. Significant induced oscillations were detected in a frequency by frequency basis with a statistical criteria that was as follows: the maximum magnitude of the oscillatory activity should be two times above the mean baseline values, in the Z-score sense. We applied this threshold for each individual contact on the hippocampus and subject (8 contacts per subject on average). We calculated the Z-score to overcome possible limitations with the baseline activity and to reduce the weight of a single patient activity. The area under the curve for the envelope of each frequency component was also computed on a trim mean of the epochs to obtain a power measure summary of the oscillatory activity.

Additionally, imaginary coherence^50^ (iCoh, measure of the linear relationship of two time-series, a conservative measure of synchronization/connectivity between contacts that has the advantage of decomposing the flow of oscillatory activity between brain regions with less influence of the volume conduction) was computed for all the contact pairs (hippocampus contacts to all the other contacts) and for each task for the whole frequency range 5–80 Hz. This was performed using the HERMES toolbox^47^ with an overlapping window of 10%, lag of 200 for all individual frequency points. The thresholded results were visualized using the BrainNet viewer network visualization tool^48^.

### Statistical analyses

We report intracranial EEG recordings from the hippocampus of epilepsy patients. TF data within each trial were normalized by converting each data point into a z-score relative to the entire trial time-series. The computed z-scores were then baseline corrected by subtracting an average of prestimulus baseline data points.

We used non-parametric statistical tests (Wilcoxon) to assess significant task modulations. We computed statistical analysis (Friedman test) comparing the power per frequency band, tasks and hippocampal regions (anterior/posterior). The alpha value was set to 0.05 and false discovery rate correction for multiple comparisons was applied whenever applicable. Moreover, to assess the correlation between task difficulty and power activity, non-parametric correlations (Spearman test) were performed between task memory load and power area-under-the-curve. Regarding connectivity, each pair of iCoh values was assessed for significance from zero by employing the commonly used method of surrogate statistics with 1000 surrogates and connectivity was plotted at a thresholded of p < 0.025. To further confirm the strength of the effects we calculated the effect size (Cohen’s d) of the power differences per task condition and these were: 0.24 for Copy Rey, 0.35 for Visuospatial Memory, 0.45 and 0.47 for the Benton Face recognition and Benton JLO respectively; 0.55 for the long term memory, 0.76 for the Recall of the Rey figure and 0.79 for the Sel.Attention. On average the effect size was d = 0.51 ± 0.2.

## Supplementary Information

Supplementary Video 1.

Supplementary Video 2.

Supplementary Figure 1.

Supplementary Legends.

## Data Availability

The datasets used and/or analysed during the current study available from the corresponding author on reasonable request.

## References

[1] Greicius MD (2003). Regional analysis of hippocampal activation during memory encoding and retrieval: fMRI study. Hippocampus.

[2] Norman Y (2019). Hippocampal sharp-wave ripples linked to visual episodic recollection in humans. Science (80-).

[3] Nadel L, Hoscheidt S, Ryan LR (2013). Spatial cognition and the hippocampus: The anterior-posterior axis. J. Cogn. Neurosci..

[4] Schacter DL, Wagner AD (1999). Medial temporal lobe activations in fMRI and PET studies of episodic encoding and retrieval. Hippocampus.

[5] Lepage M, Habib R, Tulving E (1998). Hippocampal PET activations of memory encoding and retrieval: The HIPER model. Hippocampus.

[6] Fanselow MS, Dong H-W (2010). Are the dorsal and ventral hippocampus functionally distinct structures?. Neuron.

[7] Strange B, Fletcher P, Henson R, Friston K, Dolan R (1999). Segregating the functions of human hippocampus. Proc. Natl. Acad. Sci..

[8] Kahn I, Andrews-Hanna JR, Vincent JL, Snyder AZ, Buckner RL (2008). Distinct cortical anatomy linked to subregions of the medial temporal lobe revealed by intrinsic functional connectivity. J. Neurophysiol..

[9] Grunwald T, Lehnertz K, Heinze HJ, Helmstaedter C, Elger CE (1998). Verbal novelty detection within the human hippocampus proper. Proc. Natl. Acad. Sci. U. S. A..

[10] Duncan K, Ketz N, Inati SJ, Davachi L (2012). Evidence for area CA1 as a match/mismatch detector: A high-resolution fMRI study of the human hippocampus. Hippocampus.

[11] Strange BA, Witter MP, Lein ES, Moser EI (2014). Functional organization of the hippocampal longitudinal axis. Nat. Publ. Gr..

[12] Zeidman P, Lutti A, Maguire EA (2015). Investigating the functions of subregions within anterior hippocampus. Cortex.

[13] Melani F, Zelmann R, Mari F, Gotman J (2013). Continuous high frequency activity: A peculiar SEEG pattern related to specific brain regions. Clin. Neurophysiol..

[14] Caplan JB (2003). Human theta oscillations related to sensorimotor integration and spatial learning. J. Neurosci..

[15] Axmacher N (2010). Intracranial EEG correlates of expectancy and memory formation in the human hippocampus and nucleus accumbens. Neuron.

[16] Taussig D, Montavont A, Isnard J (2015). Invasive EEG explorations. Neurophysiol. Clin..

[17] Merkow MB, Burke JF, Kahana MJ (2015). The human hippocampus contributes to both the recollection and familiarity components of recognition memory. Proc. Natl. Acad. Sci..

[18] Zheng J (2017). Amygdala-hippocampal dynamics during salient information processing. Nat. Commun..

[19] Gonzalez-Martinez J, Lachhwani D (2014). Stereoelectroencephalography in children with cortical dysplasia: Technique and results. Childs. Nerv. Syst..

[20] Cardinale F (2013). Stereoelectroencephalography: Surgical Methodology, safety, and stereotactic application accuracy in 500 procedures. Neurosurgery.

[21] Crone N, Sinai A, Korzeniewska A (2006). High-frequency gamma oscillations and human brain mapping with electrocorticography. Prog. Brain Res..

[22] Jacobs J (2012). High-frequency oscillations (HFOs) in clinical epilepsy. Prog. Neurobiol..

[23] Kucewicz MT (2014). High frequency oscillations are associated with cognitive processing in human recognition memory. Brain.

[24] Moroni F (2014). Hippocampal slow EEG frequencies during NREM sleep are involved in spatial memory consolidation in humans. Hippocampus.

[25] Bush D (2017). Human hippocampal theta power indicates movement onset and distance travelled. Proc. Natl. Acad. Sci..

[26] Duarte IC, Castelhano J, Sales F, Castelo-Branco M (2016). The anterior versus posterior hippocampal oscillations debate in human spatial navigation: Evidence from an electrocorticographic case study. Brain Behav..

[27] Castelhano J, Duarte ICIC, Wibral M, Rodriguez E, Castelo-Branco M (2014). The dual facet of gamma oscillations: Separate visual and decision making circuits as revealed by simultaneous EEG/fMRI. Hum. Brain Mapp..

[28] Castelhano J (2017). Cortical functional topography of high-frequency gamma activity relates to perceptual decision: An intracranial study. PLoS ONE.

[29] Lachaux J-P, Axmacher N, Mormann F, Halgren E, Crone NE (2012). High-frequency neural activity and human cognition: Past, present and possible future of intracranial EEG research. Prog. Neurobiol..

[30] Engel AK, Fries P, Singer W (2001). Dynamic predictions: Oscillations and synchrony in top-down processing. Nat. Rev. Neurosci..

[31] Jerbi K (2009). Task-related gamma-band dynamics from an intracerebral perspective: Review and implications for surface EEG and MEG. Hum. Brain Mapp..

[32] Wacker M, Witte H (2013). Time-frequency techniques in biomedical signal analysis. A tutorial review of similarities and differences. Methods Inf. Med..

[33] Keil A, Gruber T, Müller MM (2001). Functional correlates of macroscopic high-frequency brain activity in the human visual system. Neurosci. Biobehav. Rev..

[34] Le Van Quyen M, Bragin A (2007). Analysis of dynamic brain oscillations: Methodological advances. Trends Neurosci..

[35] Moses DA, Leonard MK, Makin JG, Chang EF (2019). Real-time decoding of question-and-answer speech dialogue using human cortical activity. Nat. Commun..

[36] Glanz Iljina O (2018). Real-life speech production and perception have a shared premotor-cortical substrate. Sci. Rep..

[37] Park H-D (2018). Neural sources and underlying mechanisms of neural responses to heartbeats, and their role in bodily self-consciousness: An intracranial EEG study. Cereb. Cortex.

[38] Koessler L (2010). NeuroImage source localization of ictal epileptic activity investigated by high resolution EEG and validated by SEEG. Neuroimage.

[39] Benton A, Hamsher KD, Varney N, Spreen O (1983). Contribution to Neuropsychological Assessment.

[40] Benton AL, Van Allen MW (1972). Prosopagnosia and facial discrimination. J. Neurol. Sci..

[41] Gauthier L, Dehaut F, Joanette Y (1989). The Bells test: A quantitative and qualitative test for visual neglect. Int. J. Clin. Neuropsychol..

[42] Corwin J, Bylsma FW (2007). Psychological examination of traumatic encephalopathy. Neuropsychologist..

[43] Corsi, P. Human memory and the medial temporal region of the brain. *Dis. Abstr. Int.* **34** (1972).

[44] Koessler L (2007). Spatial localization of EEG electrodes. Neurophysiol. Clin..

[45] Mcgonigal A (2007). Stereoelectroencephalography in presurgical assessment of MRI-negative epilepsy. Brain.

[46] Delorme A, Makeig S (2004). EEGLAB: An open source toolbox for analysis of single-trial EEG dynamics including independent component analysis. J. Neurosci. Methods.

[47] Niso G (2013). HERMES: Towards an integrated toolbox to characterize functional and effective brain connectivity. Neuroinformatics.

[48] Xia M, Wang J, He Y (2013). BrainNet viewer: A network visualization tool for human brain connectomics. PLoS ONE.

[49] Uhlhaas PJ (2006). Dysfunctional long-range coordination of neural activity during Gestalt perception in schizophrenia. J. Neurosci..

[50] Nolte G (2004). Identifying true brain interaction from EEG data using the imaginary part of coherency. Clin. Neurophysiol..

