# Supplementary figures and images for: Intracranial recordings in humans reveal specific hippocampal spectral and dorsal vs. ventral connectivity signatures during visual, attention and memory tasks
Source: Sci Rep. 2022 Mar 3;12:3488. doi: 10.1038/s41598-022-07225-0 (PMC8894428; doi:10.1038/s41598-022-07225-0)

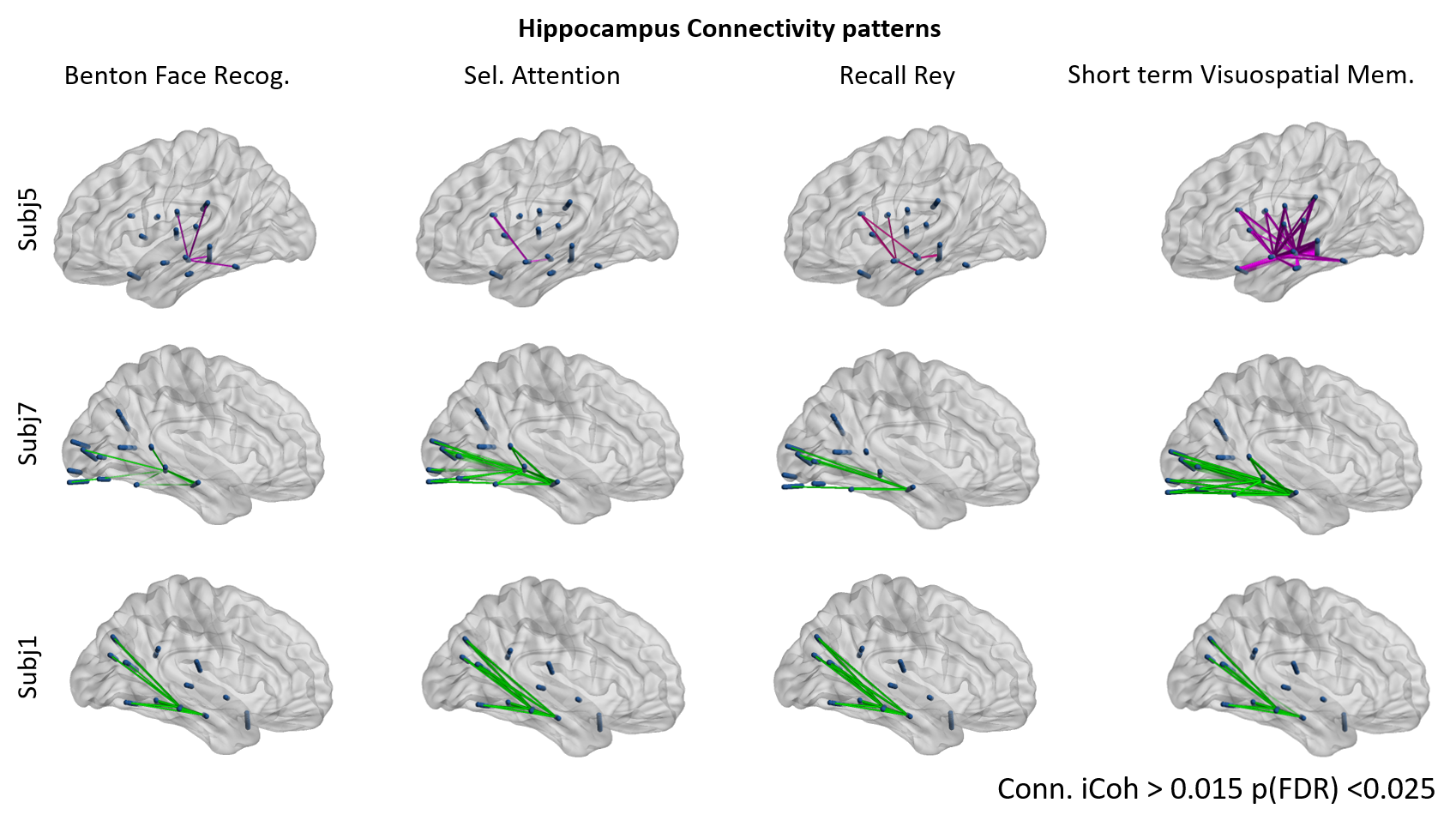

Supplement: Supplementary file 3 — Supplementary Figure 1. [file 41598_2022_7225_MOESM3_ESM.tif]
