# Supplementary material for: Intracranial recordings in humans reveal specific hippocampal spectral and dorsal vs. ventral connectivity signatures during visual, attention and memory tasks
Source: Sci Rep. 2022 Mar 3;12:3488. doi: 10.1038/s41598-022-07225-0 (PMC8894428; doi:10.1038/s41598-022-07225-0)
Supplement: Supplementary file 4 — Supplementary Legends. [file 41598_2022_7225_MOESM4_ESM.docx]

**Supplementary material 1**—example video available (patient wearing a cap and positioned to prevent identification). This real-time scenario in long recording periods provides a unique opportunity to evaluate the brain in a **real neuropsychological evaluation clinical setting,** unlike other less ecological and more rigid experimental approaches.

**Supplementary material 2—**video of those hippocampus locations.

[**Supplementary**](sps:id::sec15) **material 3—Individual connectivity plots example for three different subjects in four distinct tasks.**
